# Less trash, more treasure. Waste production and reduction in Orthopaedic surgery

**DOI:** 10.1111/ans.70018

**Published:** 2025-02-20

**Authors:** Nikolas Drobetz, Joshua Xu, David Chang, Daniel Hazan, William Collins, Herwig Drobetz

**Affiliations:** ^1^ Lismore Base Hospital 60 Uralba St Lismore New South Wales 2480 Australia; ^2^ Bond University 14 University Dr Robina Queensland 4226 Australia; ^3^ School of Medicine The University of Sydney Robina Queensland 4226 Australia

**Keywords:** environmental, orthopaedic trauma surgery, sustainability

## Abstract

**Background:**

The operating theatre generates substantial waste, raising environmental concerns. This study quantified the waste generated during four common orthopaedic procedures and identified recyclable materials. It also assessed the associated carbon footprint to highlight opportunities for sustainable waste management.

**Methods:**

This prospective study was conducted at a single regional hospital in New South Wales, Australia, from July to September 2024. Sixty procedures were analysed: 15 total knee arthroplasties (TKA), 15 total hip arthroplasties (THA), 15 ankle fracture fixations, and 15 hand injury surgeries. Waste was categorized as landfill, recyclable, or biohazardous. Landfill waste was further analysed for potentially recyclable components, with data extrapolated to nationwide operation numbers. Carbon dioxide emissions were calculated using the formula tCO_2_‐e = Q × EF, where Q is waste weight, EF is the emission factor (0.879), and tCO_2_‐e is tonnes of carbon dioxide emissions.

**Results:**

Across 60 procedures, 425.7 kg of waste was generated, averaging 8.2 kg per case. TKA produced the most waste (11.7 kg per case), while hand injury surgeries generated the least (3.9 kg per case). Potentially recyclable waste constituted 12% of landfill waste, with TKA having the highest recyclable proportion (13%). Recyclable materials comprised 44% of total waste. Proper segregation could reduce up to 75 t of CO_2_‐emissions annually from TKA alone in Australia.

**Conclusions:**

A significant portion of orthopaedic theatre waste is recyclable, offering opportunities to reduce the carbon footprint of surgeries. Improved staff training and recycling facilities are crucial for optimizing waste management in healthcare.

## Introduction

In 2017, the healthcare sector in New South Wales produced 1634 kt of waste, which was ~8% of the state's total waste production. The healthcare sector also produced 7908 kt of greenhouse gas emissions in the same year.^1^ The significant waste production from hospitals thus needs to be addressed to reduce our environmental footprint. This is also in light of Australia's commitment to reduce greenhouse gas emissions to zero by 2050.^2^

It is estimated that up to 70% of all waste in a hospital comes from operating theatres.^3^ With regard to orthopaedic surgery, joint arthroplasty has been shown in numerous studies to be the most significant contributor to waste production.^4^ Despite this, there is scarce literature published on the carbon footprint that the operating theatre produces.

Our study aimed to quantify the waste produced in the operating theatre for four common orthopaedic operations. In addition to this, we measured the amount of recyclable material in landfill waste to calculate the excess carbon dioxide emissions produced. To our knowledge, this is the first study in Australia on this topic.

## Materials and methods

### Study design

Data were prospectively collected from a single regional hospital in New South Wales. Collection of data occurred between 1 July and 18 September 2024. The two elective procedures were consecutive primary total hip and knee arthroplasties (THA, TKA), and the two trauma procedures were consecutive ankle fractures managed with open reduction and internal fixation (ORIF) and consecutive hand injuries. Hand injuries included debridement of traumatic wounds and ORIF for phalanx or metacarpal fractures. These procedures were selected as all surgeons at our centre routinely perform them, reducing potential bias compared to single surgeon data. Hand injuries were used as a surrogate for ‘small’ common operations, ankle fractures for common trauma procedures with a more or less constant amount of osteosynthesis material inserted and hip and knee arthroplasties as a surrogate for common arthroplasty.

After completion of each case, the total recyclable, landfill, and biological waste was weighed. The landfill waste was then sorted through, and any recyclable waste from the landfill was weighed again separately. For study purposes, we labelled it ‘potentially recyclable’. We did not include sharp waste products and waste produced by the anaesthetic team. The sorting of waste was completed by five staff members who all had training regarding what constitutes potentially recyclable waste (Table 1). A handheld electronic scale was used to measure all waste with precision to within 10 g.

**Table 1:** Definitions and explanation of waste types

Waste type	Definition and examples
Recycling	Material deemed to be recyclable such as hard plastic, cardboard packaging, paper, equipment tray cover, syringes†
Landfill	Material deemed to be not recyclable and was considered as general waste. Examples include soiled draping, sponges, suture material
Biological	Material that is deemed to be biological and hazardous such as heavily soiled drapes, or human tissue
Potentially recyclable	Material that was removed from the landfill and deemed to be recyclable such as hard plastic, cardboard packaging, paper, equipment tray cover, syringes†

†Not soiled by blood or patient tissue.

### CO_2_ calculation

CO_2_ (carbon dioxide) production was calculated using the following formula: tCO_2_‐e = Q × EF.^5^

tCO_2_‐e is the tonnes of carbon dioxide emissions, Q is the weight of clinical waste, and EF is the emission factor. The Australian National Greenhouse Accounts Factors state that the emission factor produced by removing clinical waste is 0.879.^5^

### Number of procedures, data extrapolation and ethics

Data collection of this study was completed for 60 operations, 15 per procedure subtype, in accordance with the data calculated by Stall *et al*.,^6^ who concluded that 10 procedures per subtype were sufficient to achieve reliable results. All of the data were recorded in Microsoft Excel 2024. The study was exempted from HREC approval as no human subjects were involved in data collection (Reference number QA520).

## Results

Total waste production from 60 procedures was 425.7 kg, with a mean of 8.2 kg (range, 3.8–15.7) per operation and a median of 8.4 kg. Total knee arthroplasty produced a mean total of 11.7 kg (range, 9.7–13.3) per case. Total hip arthroplasty produced 11.6 kg (range, 7.6–15.7) per case, with one outlier due to surgical field contamination and re‐draping of the field. The proportion of potentially recyclable waste was, however, not significantly different from all other procedures. Ankle fracture ORIF produced on average 5.9 kg (range, 4.6–7.1). Surgery for hand injuries saw a mean of 3.9 kg (range, 2.8–6) per case.

Potentially recyclable waste among landfill was a mean of 0.8 kg (range 0.1–2.1) per case. The mean percentage of potentially recyclable waste from landfill was 12.5% (range 11.9–13). Total knee arthroplasty produced the most potentially recyclable material, with a mean of 13%. For detailed results, see Table 1.

There was no statistically significant difference between total waste produced from THA and TKA (*P* = 0.42). THA and TKA produced significantly greater waste compared to ankle and hand operations (*P* < 0.001). There was also no significant difference in the potentially recyclable material as a proportion of the landfill across all operations (*P* = 0.93).

## Discussion

**Fig. 1:**
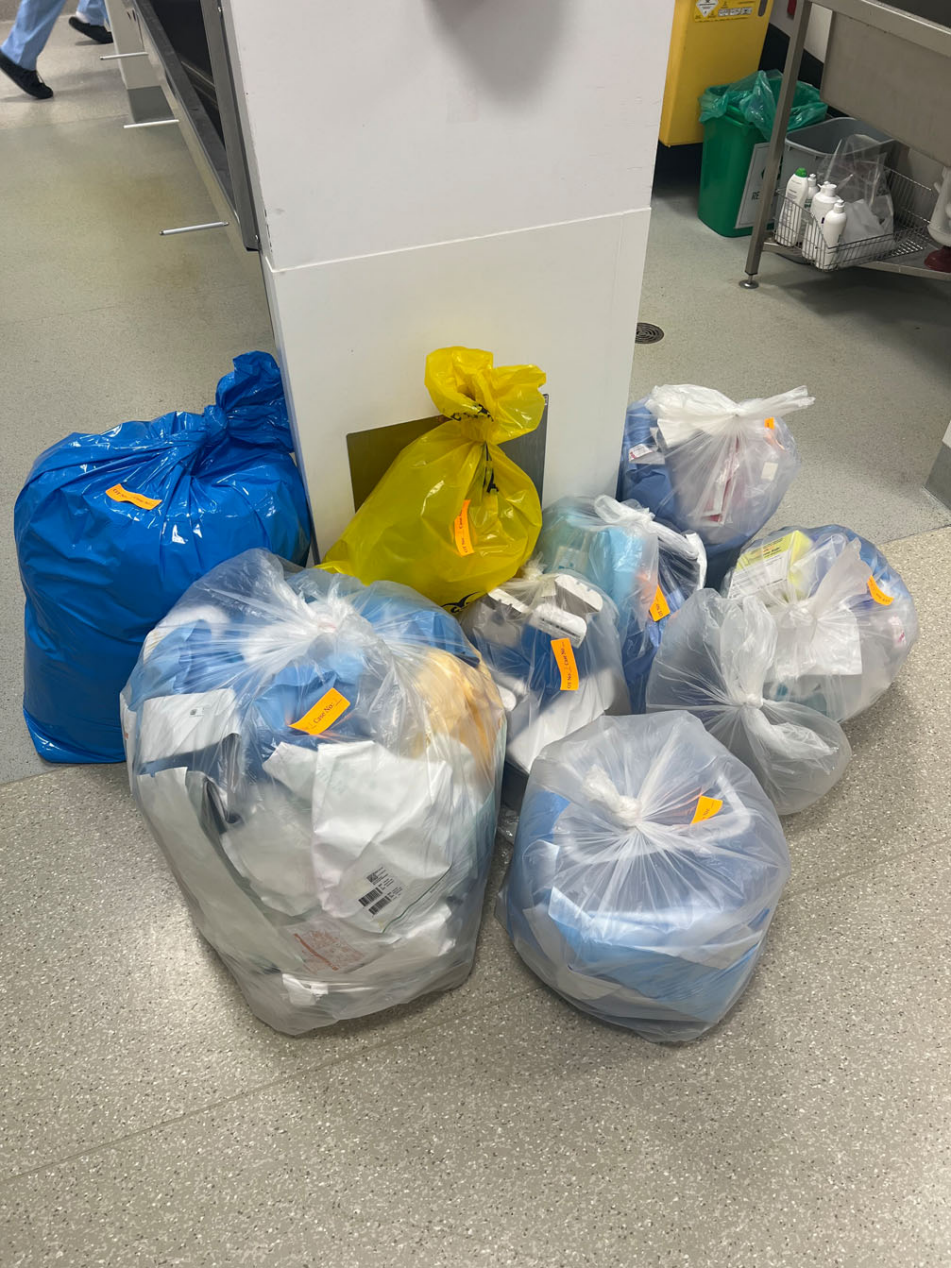
Waste collected after a total knee arthroplasty at our facility.

Our study showed that a landfill waste contained a significant amount of material that could have potentially been recycled. Potentially recyclable waste from the landfill was a mean of 80% of the amount of already recycled waste, meaning that the amount of recyclable waste can potentially almost be doubled. The highest total waste production was TKA at an average of 11.7 kg, with the lowest production being upper limb surgery at 3.9 kg. The surgeries that saw the highest and lowest amounts of potentially recyclable material as percentages were TKA and THA, respectively. Figure 1 demonstrates the waste produced after one total knee arthroplasty at our facility.

TKA had the most potentially recyclable waste at 13% of the total waste (Table 2). This is likely due to the large number of single‐packed items and hard plastics involved in this operation. Lee *et al*. showed that TKAs produced an average of 13.6 kg compared to 11.7 kg in our study. The percentage of potentially recyclable waste for THA and TKA in their study was 22.8% and 22%, respectively.^7^ This is ~10% more than what our study found, which may be due to different waste practices and definitions of recyclable waste. Data from our centre suggests we are not maximizing our recycling from theatre, as our recycling stream on average is 25% (Table 2). Other studies have reported up to 40% of their total waste stream being recyclable.^8^ This may be due to difference in definitions of recyclable material along with differences in packaging, tray set up, resources, and staff training. The current practice at our centre is not standardized, and theatre waste is not routinely separated into biohazard, landfill, and recyclable waste.

**Table 2:** Waste production of standardized orthopaedic procedures

Operation	Total waste produced (kg)	Mean total waste (kg)	Mean landfill (kg)	Total recycling (kg)	Mean recycling (kg)	Mean biohazard (kg)	Total pot. recyclable (kg)	Mean total pot. recyclable (kg)	Mean percentage of pot. recyclable of landfill (%)	Recycling and pot. recycling total (kg)	Pot. recycling as a percentage of all recycled material (%)	Pot. recycling and recycling as a percentage of total waste (%)
Hand injuries	58.13	3.88 (2.79–5.99)	3.24 (2.69–4.11)	7.57	0.50 (0.1–1.33)	0.13 (0–0.55)	5.98	0.4 (0.23–0.57)	12.34	13.55	44.13	23.30
Ankle	82.11	5.87 (4.6–7.12)	4.67 (3.5–6.26)	12.56	0.89 (0.3–1.57)	0.3 (0–0.87)	8.28	0.59 (0.1–1.42)	12.63	20.84	39.73	25.38
TKA	162.33	11.68 (9.73–13.29)	9.61 (11.6–7.2)	22.21	1.48 (0.24–3.36)	0.59 (0–1.9)	18.75	1.25 (0.8–2.11)	13.01	40.96	45.78	25.23
THA	161.79	11.56 (7.6–15.73)	9.45 (7.6–11.3)	19.95	1.22 (0.4–4.43)	0.56 (0–1.25)	16.89	1.13 (0.6–1.72)	11.96	36.84	45.84	22.77

The three major waste streams in theatre were landfill, recycling, and biological waste. Throughout the completion of our study, we found that there was a significant difference in how staff disposed of waste – often, recyclable material was placed into landfill containers and vice versa. The reason for this may include improper knowledge of what material in theatre is recyclable. Azouz *et al*. found that 47.7% of operating staff believe poor knowledge of recyclables is the most significant barrier to recycling in theatre.^9^ By conducting training for all staff, a reduction of potentially recyclable waste incorrectly placed in landfill may be seen. Another reason there is variation in waste disposal may be the inadequate availability of recycling bins throughout operating theatres. Improving waste management at our centre may be as simple as placing a large and clearly labelled recycling bin in all operating theatres. We have developed a simple guideline for staff education, see supplemental material (Data S1). While not within the scope of this study, it is essential to note that waste reduction should also start at the front end by aiming to produce less waste in the beginning, for example, avoid single‐packed or single‐use items as much as possible.

Balch *et al*.^10^ completed a scoping review looking at the reduction of waste production in theatre. They found that the most significant reduction of waste was instrument tray optimisation. An example of this may be to only open instrument trays which have a considerable chance of being used in an operation. Our study also found that instrument tray wrapping was often not recycled, although it contributed significantly to the potentially recyclable waste. If recycled properly, the polypropylene in blue wrap can be turned into medical devices.^11^ Further, companies producing modern orthopaedic devices and technology often overpack their equipment. Examples of this include multi‐page instructions on single‐use screws with a large hard plastic cover. Hospitals should insist that these companies modify their packaging to reduce the waste production of these items.^6^

We extrapolated the data for all four‐operation types to highlight the sheer volume of waste production in orthopaedic procedures (Table 3). For simplicity, we will discuss only TKA data (Table 3). In 2021, a total of 68 466 total knee arthroplasties were performed in Australia.^12^ Using our data would mean that ~800 000 kg of total waste is produced. This would also mean that ~85 600 kg of waste in total knee arthroplasties could be potentially recyclable material in Australia alone. Using data reported by the Australian Government, we can estimate that proper waste segregation could save 75 t of CO_2_ emissions going into the atmosphere per year by identifying potentially recyclable material.^5^ This is the equivalent of driving a regular passenger car for 512 000 km (146.5 g of CO_2_ emissions per km).^13^ The healthcare sector contributes 7% of Australia's total carbon dioxide emissions, meaning a national healthcare effort to reduce emissions could significantly impact Australia's global carbon footprint.^14^ Training, education, and improved resources in hospitals across Australia can reduce these emissions and improve our impact on greenhouse gases.

**Table 3:** Average weight of different waste streams and CO_2_ production extrapolated to Australian data^5^, ^12^, ^20^, ^21^

Waste stream	Mean mass (kg)	Extrapolated weight (kg)†	Extrapolated CO_2_ production (tonnes)†
**Hand injuries**			
Landfill	3.2	106 920	94
Recyclable	0.5	16 500	14.5
Biohazard	0.1	4290	3.8
Potentially recyclable	0.4	13 200	11.1
**Ankle**			
Landfill	4.7	44 365	39.9
Recyclable	0.9	8455	7.5
Biohazard	0.3	2850	2.5
Potentially recyclable	0.6	5605	4.9
**TKA**			
Landfill	9.6	658 285	578.4
Recyclable	1.5	101 380	89.1
Biohazard	0.6	40 415	35.5
Potentially recyclable	1.3	85 626	75.2
**THA**			
Landfill	9.5	498 960	438.5
Recyclable	1.2	64 416	56.6
Biohazard	0.6	29 568	26
Potentially recyclable	1.1	59 664	52.4
Totals (excluding potentially recyclable)		1 576 404 kg	1386 t

†The data are an estimate, exact values will vary.

Environmental sustainability in hospitals should not be restricted to operating theatres. Successful waste reduction relies on creating an environmental stewardship team,^6^ with initiatives such as bins dedicated to PVC lines and piping, donation of expired medical equipment to veterinarians, composting food scraps, and re‐using hard plastic equipment trays.^15^ In 2020, this initiative saw the Queensland Children's Hospital recycle 357 000 kg of waste and compost 11 t of food scraps.^16^ Further initiatives such as the ‘5‐Rs’ rule: Reduce, Reuse, Recycle‐Renew, Rethink, and Renewables energies proposed by Pradere *et al*.^17^ can help reduce our carbon footprint in healthcare. Implementation of strategies such as this across health services in Australia could make a significant impact on environmental sustainability, as well as save substantial costs on waste disposal. On a global scale, initiatives like the Lancet Countdown on Health and Climate Change try to raise awareness for health transformation.^18^ Furthermore, awareness of environmental sustainability should be included in the training for surgeons worldwide.^19^

### Limitations

There are several limitations to our study. First, our study was only completed at a single centre, so may not represent the waste collection process of other hospitals in Australia. Second, surgeon preference and, therefore waste production varies, depending on the operation performed. The surgeon's experience and training may also affect waste production for each operation. To counteract this potential bias, we chose common operations as mentioned above and the waste produced in each category showed only minor variations. The other reason for choosing common procedures was that they are more reflective of standard practice in a regional setting. Evaluating less common operations would take a disproportionate amount of time but would not contribute significantly to the overall results. Non‐routine procedures also produce more waste as staff opens more trays and implants and instruments need to specifically be ordered. We wanted to show a ‘lowest common multiple’ and felt that the best way to achieve this is by choosing common operations.

Regarding procedure numbers, we decided to include 15 rather than 10 procedures per subtype as suggested by Stall *et al*.^6^ and increase the numbers in case waste production would show significant deviation between cases. It became evident early in the process, however, that the proportion of recyclable waste was constant with non‐significant differences and also that the absolute waste amount did not significantly differ between cases. We therefore think that adding more operations will not add additional knowledge. It would be interesting, however, to study larger numbers by means of a multi‐centre study, also to see inter‐hospital differences.

## Conclusion

Our study showed that the amount of recyclable waste can potentially nearly be doubled. The average potentially recyclable waste produced from our study was 12.5%. This number is concerning for its environmental impact, given the large number of orthopaedic procedures done each year in Australia. Our study identified a vast and immediate potential to improve how we manage and deal with waste produced in surgery. Different strategies can be implemented to increase our potentially recyclable waste. The first strategy includes staff training and education on what defines recyclable material and how we can dispose of it correctly. Another approach comprises placing large, well‐marked recycling bins in theatre. In addition to this, visual aids in the form of a poster can be used to help reduce confusion and promote correct recycling. Incentive‐based challenges to promote recycling and reusage could be another potential strategy. Future studies should evaluate the difference in potentially recyclable material in theatre waste after a period of implementing changes such as this.

## Supporting information


**Data S1.** Supporting Information.
