# Supplementary material for: Less trash, more treasure. Waste production and reduction in Orthopaedic surgery
Source: ANZ J Surg. 2025 Feb 20;95(3):539–43. doi: 10.1111/ans.70018 (PMC11937738; doi:10.1111/ans.70018)

FIVE

LESS TRASH, MORE TREASURE

WAYS TO REDUCE AND RECYCLE

1 APPROPRIATE DISPOSAL

use allocated bins shown on next page for proper disposal of landfill, recyclables and biohazard

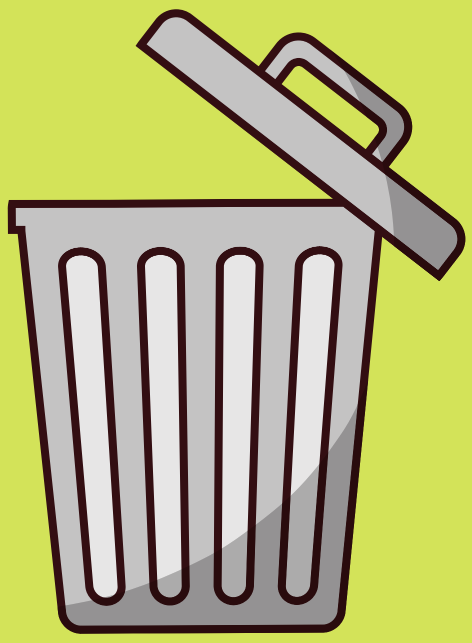

2 WHAT TO RECYCLE?

- Packaging of sterile trays
- Surgical gear packaging
- Gown tabs
- Hard plastics
- PVC piping
- Paper and cardboard

3 UNOPENED TRAYS

Tray of equipment that may or may not be used, should be spared in order to reduce producing of unecessary waste

4 WHAT TO REUSE?

Emphasis on reusable items is important. Examples include sterile blue cloths found in gown packaging

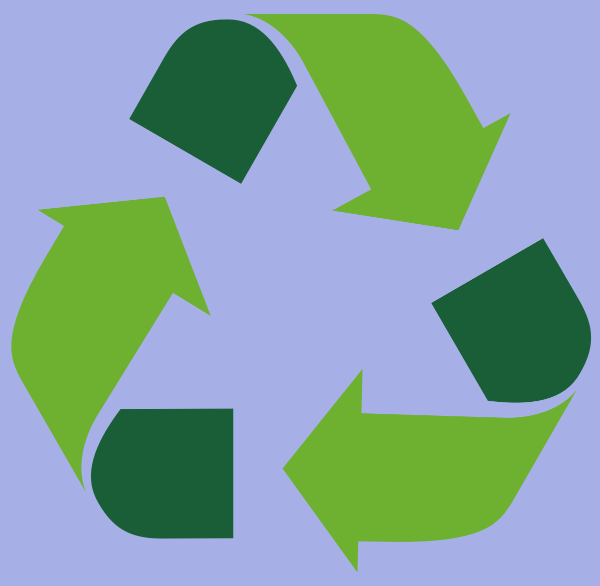

5 EDUCATION AND AWARENESS

Studies show that 50% of theatre staff beleive improper knowledge is a major factor to poor waste management. Continued awareness of this topic is vital

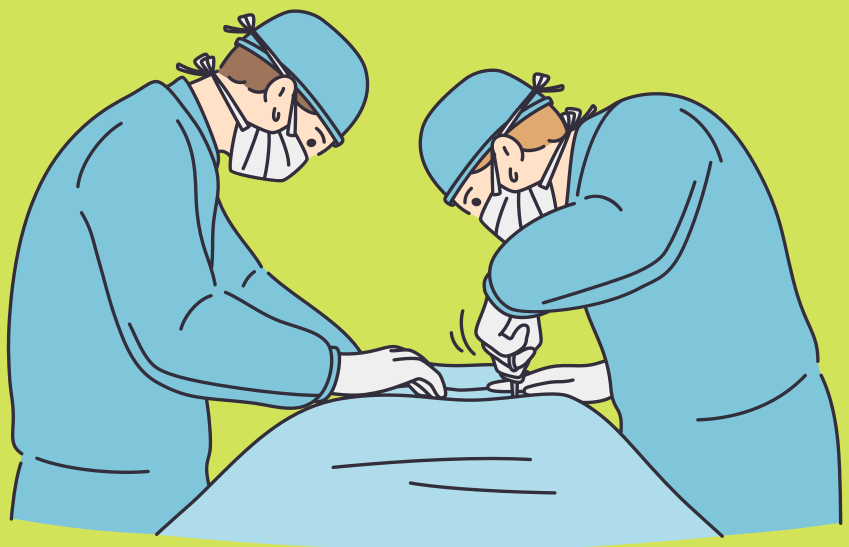

WASTE MANAGEMENT

RECYCLING AND PVC PIPING

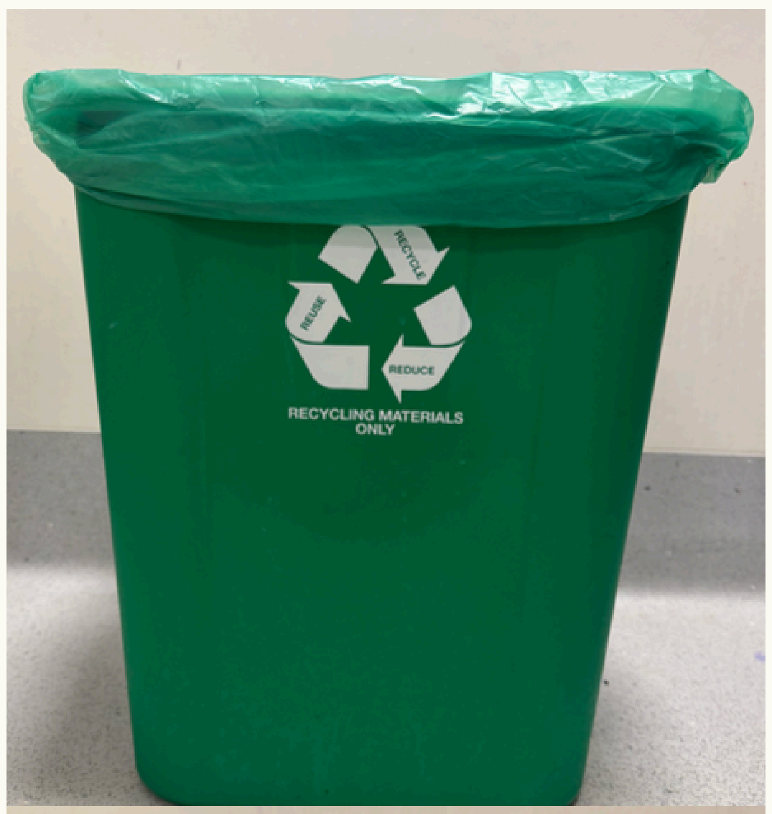

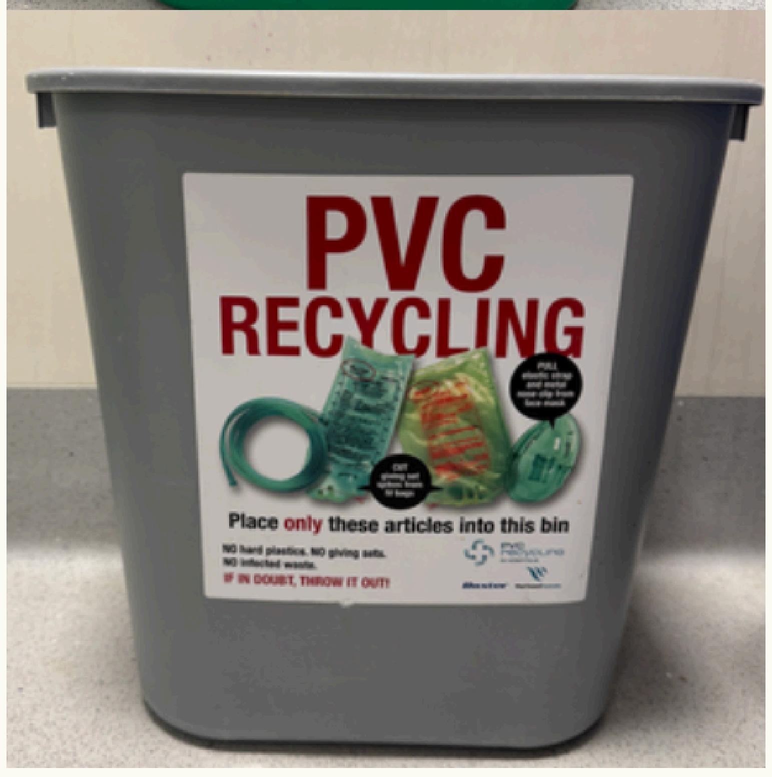

BIOHAZARD

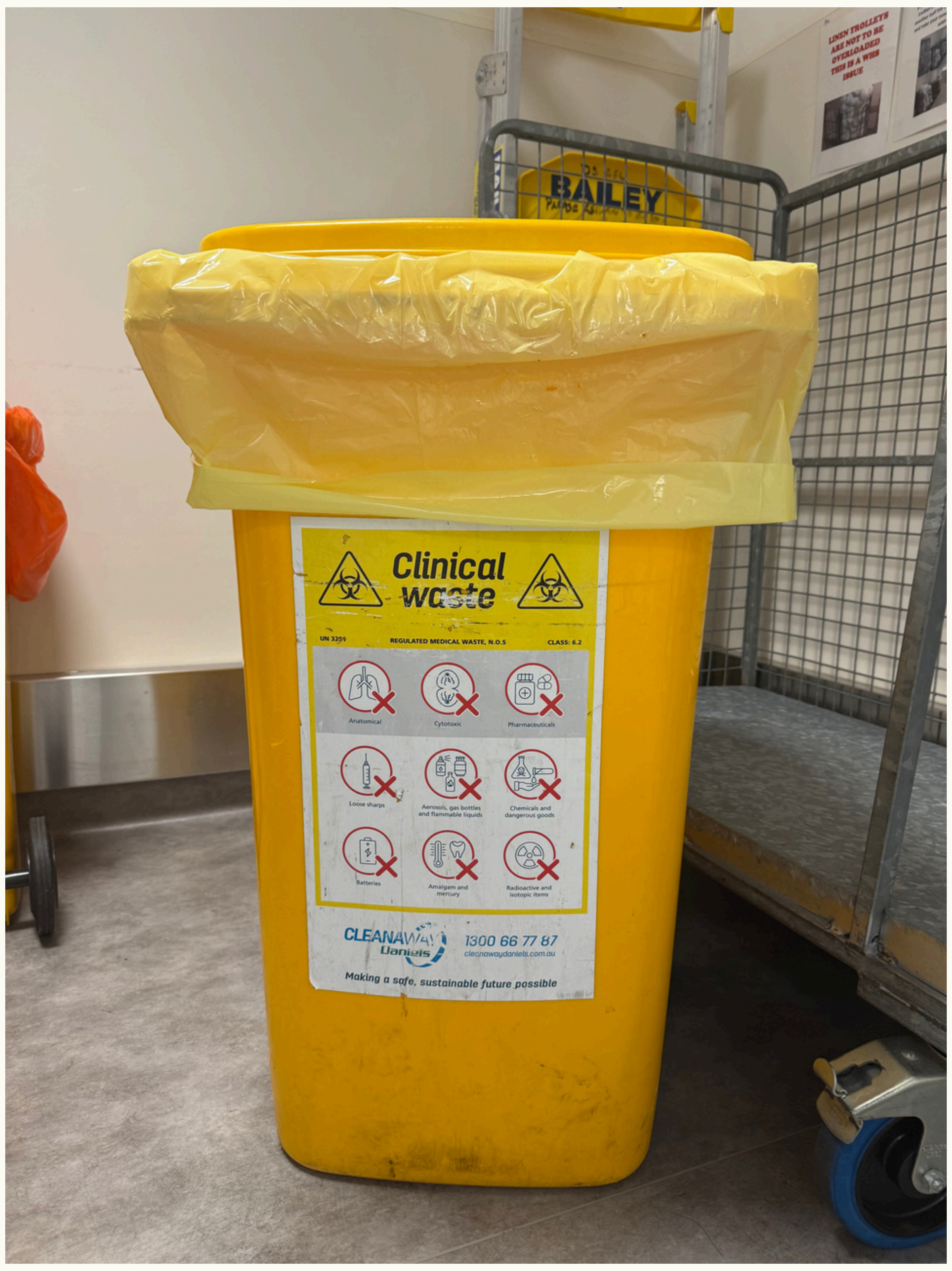

LANDFILL

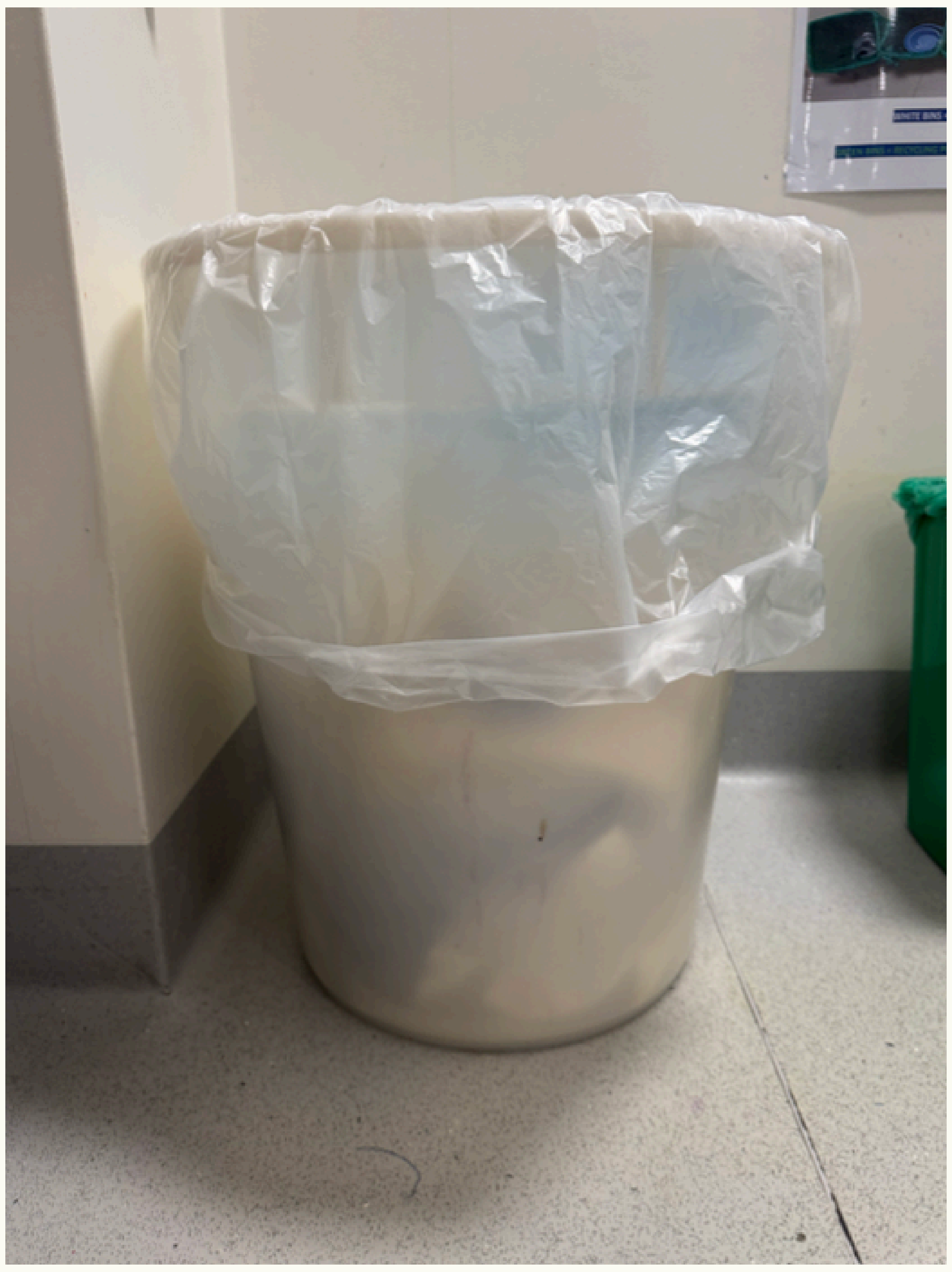

Supplement: Supplementary file 1 — Data S1. Supporting Information. [file ANS-95-539-s001.pdf]
